# Patience and its relationship to Stress Tolerance in the medical system in Bethlehem Governorate during the Corona pandemic

**DOI:** 10.1192/j.eurpsy.2023.1696

**Published:** 2023-07-19

**Authors:** N. S. Al-Arja

**Affiliations:** Social Sciences, Bethlehem University, Bethlehem, Palestinian, State of

## Abstract

**Introduction:**

The COVID-19 pandemic has set unprecedented demand on the medical system globally. Palestine was one of the Arab countries affected

**Objectives:**

The present study aims to identify the relationship between patience and Stress Tolerance in the medical system in Bethlehem Governorate during the Corona pandemic, as well as to identify the impact of several demographic variables on it.

**Methods:**

Descriptive method were used. Appropriate statistical analyses were conducted using (SPSS). A random sample of 160 workers of the medical staff completed the Patience Scale and Coping Processes Scale questionnaire.

**Results:**

: showed there is a significant positive effect for patience on stress tolerance and there were statistically significant differences in the level of patience in favor of males and single workers. It was also found that there is no difference in the specific duties of a health care worker and no variance of statistical evidence was found in the level of patience due to work with Covid patients but there were differences in stress tolerance in favor of those who do not work with Covid patients.

There is a significance in the level of stress tolerance in favor of the National Center in regard to bearing pressure.

There was a negative correlation with statistical significance between stress tolerance and age. The nature of stress changes with age, from episodic to chronic, which in turn affects appraisal and coping processes

**Conclusions:**

This study, which was conducted on a sample of Palestinian medical workers in the Bethlehem area, showed that at the beginning of the pandemic, the medical system in Palestine was not ready to handle Covid 19, and had no precautions to prevent the disease. In spite of that, the doctors and nurses who were undergoing psychological pressure were able to stand at the front line and face the outbreak of the Corona virus.

**Disclosure of Interest:**

None Declared

